# Label-free and isobaric tandem mass tag (TMT) multiplexed quantitative proteomic data of two contrasting rice cultivars exposed to drought stress and recovery

**DOI:** 10.1016/j.dib.2018.12.041

**Published:** 2018-12-15

**Authors:** Yunqi Wu, Mehdi Mirzaei, Brian J. Atwell, Paul A. Haynes

**Affiliations:** a Department of Molecular Sciences, Macquarie University, North Ryde, NSW 2109, Australia; b Department of Biological Sciences, Macquarie University, North Ryde, NSW 2109, Australia

## Abstract

Two rice cultivars, IAC1131 (drought tolerant) and Nipponbare (drought sensitive), with contrasting genetic backgrounds and levels of tolerance to drought, were analysed using both label-free and tandem mass tags (TMTs) quantitative proteomics approaches, aiming to elucidate the mechanisms of drought tolerance. Four-week-old seedlings of both cultivars were grown in large soil volumes in the glasshouse under controlled conditions and then exposed to moderate and extreme drought for 7 days, followed by 3 days of re-watering period. Mature leaves were harvested from plants of each treatment for protein extraction and subsequent complementary shotgun proteomic analyses. The data from this study are related to the research article “Quantitative proteomic analysis of two different rice varieties reveals that drought tolerance is correlated with reduced abundance of photosynthetic machinery and increased abundance of ClpD1 protease” (Wu et al., 2016) [1].

## Specifications table

**Table:** 

Subject area	Biology
More specific subject area	Label-free and Tandem Mass Tags (TMTs) quantitative proteomics data of rice leaves exposed to drought stress and recovery
Type of data	Figure, Supplementary Tables
How data was acquired	SDS PAGE and mass spectrometry normalized data/TMT labeled peptides and mass spectrometry normalized data
Data format	Raw, analyzed
Experimental factors	Contrasting cultivars (tolerant and sensitive), drought stress, extreme drought, moderate drought, recovery period, leaf tissue
Experimental features	Rice seedlings were exposed to drought stress and leaf proteome were analyzed by label free or TMT quantitative proteomics.
Data source location	Sydney, NSW, Australia
Data accessibility	Data is available within this article and via
	https://www.ebi.ac.uk/pride/archive/projects/PXD004096
	https://www.ebi.ac.uk/pride/archive/projects/PXD004118

## Value of the data

• Global protein expression of two genotypes of rice exposed to drought stress provides a deeper functional understanding of drought stress response in rice plants.
• Further analysis of these data sets may reveal novel candidate marker proteins that can provide targets for selective breeding programs in rice.
• A large scale, biologically important global proteomic analysis performed in triplicate using both label-free quantitation and TMT isobaric labelling provides an ideal platform for detailed methodological and statistical comparison studies.

## 1 Data

Total protein was extracted from the leaves of rice genotypes IAC1131 and Nipponbare that were subjected to different drought conditions. Label-free and TMT multiplexed quantitative proteomic approaches were used to obtain global protein expression profiles of two contrasting rice genotypes. The utility to the community of this large-scale dataset is two-fold; it provides a valuable source of novel candidate drought stress biomarker proteins which are of potential value in future selective breeding programs; and it also provides a significant resource for comparative analysis of results obtained using TMT isobaric labelling and label-free quantitation by spectral counting.

## 2 Experimental design, materials and methods

Data shown here evaluate the drought responses of two contracting rice genotypes, IAC1131 (drought tolerant) and Nipponbare (drought sensitive) at the proteome level.

### 2.1 Plant material, sampling and protein extraction

Rice genotypes – Nipponbare and IAC1131 – were sown in the same polyvinyl chloride (PVC) pipes lined with plastic bags. Triplicate of 5 treatments were considered in this study which include control A, moderate drought, extreme drought, recovery and control B [1]. The experiments were carried out in glass-houses with temperature set to 28/22 °C (day/night) and a 12-h photoperiod with the light intensity exceeded 700 µmol m^-2^ s^-1^ throughout. Plant seedlings were well watered for 40 days. Extreme drought stress was imposed by withholding water for 7 days. For the moderate drought treatment, the amount of water transpired by plants was recorded daily by measuring the weight of pots, and then the plants were watered with ½ of the water that had transpired. Severely stressed plants (47 days old) were re-watered to field capacity for three days. Thus, five group of triplicate samples were collected at 40 days (control A), 47 days (extreme and moderate drought) and 50 days (recovery and control B). Leaf tissue collected after each treatment was immediately lyophilized. Proteins were extracted from 50 mg of freeze-dried leaf powder using 0.07% β-mercaptoethanol and 10% trichloroacetic acid in acetone as described previously [2], followed by reduction and alkalization with 5 mM DTT and 10 mM iodoacetamide. Methanol-chloroform precipitation was then performed as described previously [3]. The resulted pellet was air-dried and solubilized in 80 µl of buffer consisting of 8 M urea in 100 mM Tris–HCl (pH 8.8). The concentration of protein in the solution was measured by BCA assay (Thermo).

### 2.2 Label free shotgun proteomic analysis of leaf samples

**Fig. 1:**
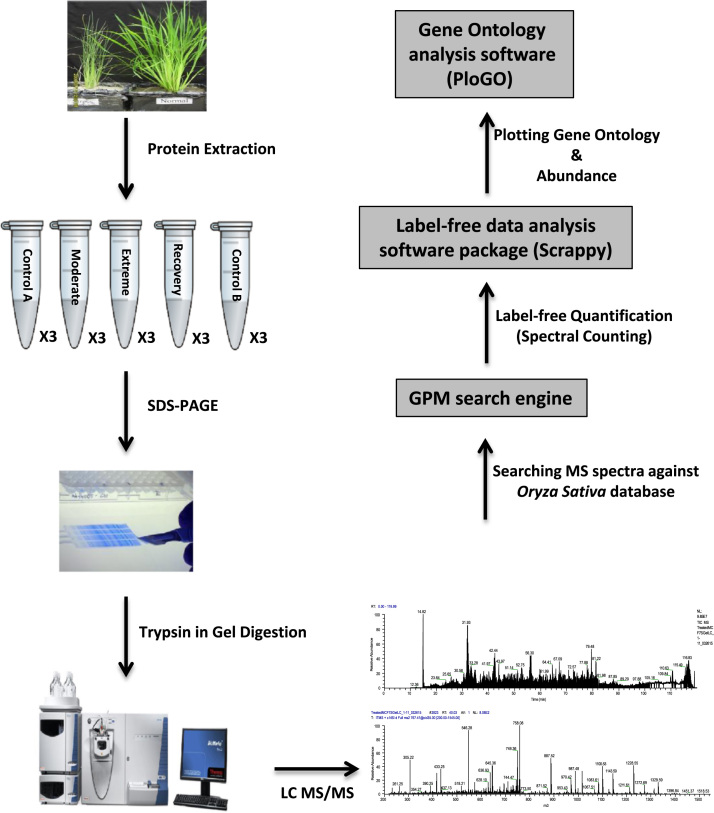
Workflow of label free quantitative shotgun proteomic analysis. Proteins extracted from rice leaves were separated by SDS-PAGE. After trypsin digestion, the resulting peptides were analysed by nanoLC-MS/MS on an LTQ-XL linear ion trap mass spectrometer. The raw files acquired from the mass spectrometer were converted into mzXML files and searched against the rice database using the GPM software. The outputs from the GPM search were further processed using the Scrappy software package to calculate normalized spectral abundance factors. The Gene ontology (GO) annotation of differentially expressed proteins was extracted from the UniProt database and matched to the list of reproducibly identified proteins using PloGo.

Proteins in urea sample buffer were fractionated into 16 fractions by sodium dodecyl sulphate-polyacrylamide gel electrophoresis (SDS-PAGE). In-gel digestion was performed as described previously [2]. The resulting peptides were analysed by nanoflow LC-MS/MS (nanoLC-MS/MS) using a LTQ-XL ion-trap mass spectrometer (Thermo, CA, USA). Peptides were separated using Magic C18AQ reversed phase columns (100 µm i.d. × 80 mm, 200 Å pore size, 3 µm bead size), with a gradient from 0–50% buffer A (5% v/v CAN, 0.1% formic acid) to buffer B (95% v/v ACN, 0.1 v/v formic acid) over 58 min. Spectra were scanned over the range 400–1500 amu with dynamic exclusion window set to 90 s and MS/ MS of the top six most intense precursor ions performed by CID at 35% normalized collision energy. The LTQ-XL raw output files were converted into mzXML format and searched against the NCBI *Oryza sativa* protein database using the global proteome machine (GPM) software (version 2.1.1) and the X!Tandem algorithm. The output from the GPM software were further processed using the Scrappy software package [4] which combines biological triplicates into a single list of reproducibly identified proteins. These proteins were retained as a valid hits in the final dataset as they were identified in all three replicates of at least one condition, and the total number of spectral counts in triplicates of at least one condition was a minimum of six [2]. Protein and peptide false discovery rates (FDRs) were calculated using reversed database searching as previously described [1]. The Scrappy program was used to calculate normalized spectral abundance factors for each protein, and perform a series of t-tests to find the proteins significantly changed in abundance between conditions [5]. The Gene ontology (GO) annotation of differentially expressed proteins was extracted from the UniProt database and matched to the list of reproducibly identified proteins using PloGo [6]. The protein identification and quantitation data for all differentially expressed proteins, along with gene ontology analysis, is presented in Supplementary Data Table 1. The workflow of label free shotgun proteomics can be viewed in Fig. 1. The mass spectrometry proteomics data have been deposited to the ProteomeXchange Consortium [7] via the PRIDE partner repository with the dataset identifier PXD004096.

### 2.3 Tandem mass tags (TMT) labeling proteomic analysis of leaf samples

**Fig. 2:**
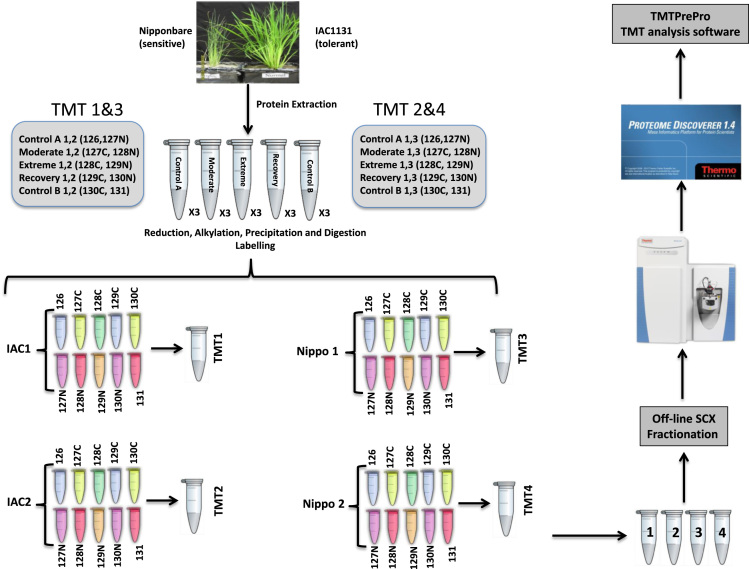
Workflow of TMT quantitative shotgun proteomic analysis. Proteins extracted from rice leaves were reduced, alkylased, precipitated and digested before being labelled with Tandem Mass Tags. Labelled samples were pooled, fractionated by SCX and then analysed on a Q Exactive Orbitrap mass spectrometer. Raw data generated from the mass spectrometer were processed using Proteome Discover v1.4 and Mascot. The search results were further analysed by the TMTPrePro software package.

Protein extracts (see Section 2.1) resuspended with 2% SDS in 50 mM Tris–HCl (pH 8.8) were digested in solution as described previously [1]. TMT labelling was performed on the resulted peptides with TMTs with respective reporter ions at *m*/*z* = 126, 127N, 127C, 128N, 128C, 129N, 129C, 130N, 130C and 131 according to the manufacturer׳s instructions. The labeled samples were pooled, desalted by solid-phase extraction before fractionation by high pressure strong cation exchange chromatography. These fractions were desalted using C18 OMIX^®^tips (Agilent) and analysed on a Q Exactive Orbitrap mass spectrometer (Thermo Scientific) coupled to an EASY-nLC1000 (Thermo Scientific). Reversed-phase chromatographic separation was carried out using a C18 HALO column, (75 μm id. × 100 mm, 160 Å pore size, 2.7 μm bead size,). Peptides were separated using a linear gradient of 1–30% solvent B (99.9% ACN/0.1% FA) over 170 min. The mass spectrometer switched between Orbitrap MS and ion trap MS/MS acquisition: full scan MS spectra (*m*/*z* 350 to 1850) were acquired at precursor isolation width of 0.7 *m*/*z*, resolution of 70,000 at *m*/*z* 400 and an AGC (Automatic Gain Control) target value of 1 × 10^6^ ions. The top ten most intense ions were fragmented by higher energy collisional dissociation (HCD) fragmentation at 35%, with dynamic exclusion for 90 s. The lock mass option was enabled using a polydimethylcyclosiloxane ion as an internal calibrant (*m*/*z* 445.12003). Raw data files generated by Xcalibur software (Thermo Scientific) were processed using Proteome Discoverer v1.4 (Thermo Scientific) and a local MASCOT server (version 2.3; Matrix Science, London, UK). The MS/MS spectra were searched against the NCBI *Oryza sativa* protein database. The mass spectrometry proteomics data have been deposited to the ProteomeXchange Consortium via the PRIDE partner repository with the dataset identifier PXD004118 [7]. The output from the Proteome Discoverer software were further processed using the TMTPrepPro package which is accessed via a local GenePattern server. Fig. 2 shows the workflow of the TMT experiments and the data from the TMTPrepPro analysis are presented in Supplementary Data Table 2.

## Appendix A Supplementary material

Supplementary material Supplementary Data Table 1. Protein identification and quantitation data for all differentially expressed proteins under moderate drought, extreme drought and recovery, along with gene ontology analysis. The Gene ontology (GO) annotation of differentially expressed proteins was extracted from the UniProt database and matched to the list of reproducibly identified proteins using PloGo. The NSAF abundance data were merged and plotted to obtain an image of overall protein abundance change over time for biological process categories.

Supplementary material Supplementary Data Table 2. TMTPrePro analysis of differentially expressed proteins under different stress conditions found in analysis of IAC1131 and Nipponbare under moderate drought, extreme drought and recovery, using TMT isobaric labelling. The thresholds for up-regulated ratio and down-regulated ratio are 1.5 and 0.66, respectively, which are highlighted with pink and green colour accordingly.
