# Supplementary material for: Label-free and isobaric tandem mass tag (TMT) multiplexed quantitative proteomic data of two contrasting rice cultivars exposed to drought stress and recovery
Source: Data Brief. 2018 Dec 15;22:697–702. doi: 10.1016/j.dib.2018.12.041 (PMC6329202; doi:10.1016/j.dib.2018.12.041)
Supplement: Supplementary file 1 — Supplementary material [file mmc1.docx]

**Manuscript No.: DIB-D-18-00809
Title: Label-free and isobaric tandem mass tag (TMT) multiplexed quantitative proteomic data of two contrasting rice cultivars exposed to drought stress and recovery
Yunqi Wu, Mehdi Mirzaei, Brian J. Atwell, Paul A. Haynes**

Conflict of interest statement.

The authors have no conflict of interest to declare in regards to this manuscript.
